# Evaluation of the Ejection Pressure for Tracking Internal Cracks during Compaction in Bilayer Tablet Formulations Using Experimental and Finite Element Methods

**DOI:** 10.3390/ph17030330

**Published:** 2024-03-02

**Authors:** Sun Ho Kim, Su Hyeon Han, Jong-Seok Oh, Dong-Wan Seo, Myung Joo Kang

**Affiliations:** 1 College of Pharmacy, Dankook University, 119, Dandae-ro, Dongnam-gu, Cheonan-si 31116, Republic of Korea; dwseomb@dankook.ac.kr (D.-W.S.); kangmj@dankook.ac.kr (M.J.K.); 2 Department of Mechanical Engineering, Kongju National University, 1223-24, Cheonan-daero, Seobuk-gu, Cheonan-si 31080, Republic of Korea; tngus8581@gmail.com; 3 Department of Future Convergence Engineering, Kongju National University, 1223-24, Cheonan-daero, Seobuk-gu, Cheonan-si 31080, Republic of Korea; jongseok@kongju.ac.kr

**Keywords:** bilayer tablet, delamination, ejection pressure, FEM, X-ray microcomputed tomography

## Abstract

This study aimed to evaluate the ejection pressure and the correlation of the findings with the occurrence of internal cracks within bilayer tablets (BLTs) consisting of metformin HCl (MF) and evogliptin tartrate (EG). Then, the mechanism of tablet failure was provided by the finite element method (FEM). The ejection pressure and the difference in diameter depending on MAIN-P were evaluated to understand the correlation between ejection pressure and change in the BLT internal structure. The ejection pressure and the difference in diameter increased as the MAIN-P increased, then steeply decreased from 350 MPa to 375 MPa of MAIN-P, despite there being no pattern in compaction breaking force and porosity. The mechanical integrity at the BLT interface was weakened by internal cracks, reducing ejection pressure. The stress distribution analysis during the compression revealed that crack formation caused by entrapped air located at the center of the BLT interface may not propagate due to concentrated stress, which promotes a tight bond at the edge of the BLT. Furthermore, complete delamination can occur in the ejection process due to localized and intensive shear stresses at the BLT interface. These findings indicate that the mechanisms of internal cracking and delamination were successfully confirmed by FEM simulation. Moreover, measuring ejection pressure before BLT manufacturing can prevent invisible tablet cracks without damaging the tablets.

## 1. Introduction

The pharmaceutical industry has achieved remarkable progress in tablet manufacturing, with significant advancements in the various types of bilayer tablet (BLT) manufacturing processes, including those of dual release tablets, barrier layer tablets, floating tablets, and multi-layer tablets.

BLTs provide many advantages over conventional monolithic tablets. The separated layer can obstruct the chemical interaction between the two APIs [1,2]. Furthermore, through the process of multi-layer compression, extended- and immediate-release profiles can be attained simultaneously in a fixed-dose combination (FDC) [3,4]. The multiple APIs with different release profiles can effectively provide complex therapeutic regimens. However, due to the complicated, multi-step process involving high-compression processes, several challenges must be overcome, including insufficient hardness [5], inaccurate individual mass control [6], cross-contamination between the layers, reduced yield, and delamination tendency at the interface between the two layers [5]. During the manufacturing and coating processes, a variety of tablet defects may occur, including chipping, cracking, sticking, and picking, as well as capping. The successful scale-up of a new drug formulation from development to manufacturing is contingent upon avoiding scale-up problems that can give rise to these tablet defects. High compression pressure increases friction at the die wall interface, leading to delamination within the tablets [7]. For highly viscoelastic powder materials, internal cracks are a common failure mode under high-strain compression [8]. These cracks might be invisible externally, being undetectable by visual inspection examinations, and potentially can increase the risk of critical failure, including degraded product quality and potential issues with disintegration and dissolution during downstream processing [7].

To investigate for invisible defects, different non-contact/non-destructive techniques have been proposed, including near-infrared spectroscopy [9,10], Raman spectroscopy [11,12], air-coupled acoustic techniques [13], terahertz pulsed imaging [14], and nuclear magnetic resonance (NMR) imaging [15]. Nevertheless, these non-invasive techniques are not feasible in pharmaceutical manufacturing because of the costly and bulky equipment, and prolonged scan durations for analysis [16]. Therefore, novel approaches that can track internal cracks in BLTs are required.

As a novel tool for tracking invisible cracks, we hypothesized that ejection pressure could be employed in the production of BLTs. Ejection pressure, which measures the frictional resistance to the tablet movement during ejection, emerges as a result of the tablet’s internally stored elastic energy causing the tablet body to elastically relax and consequently pushing onto the die walls during ejection [17]. The tablet ejection pressure was reported to depend mainly on tablet diameter, die wall friction, and residual die wall stress upon ejection [7]. To achieve successful large-scale tablet manufacturing, it is essential that the force required for ejecting the tablet from the die after compaction should not be too high [18]. Therefore, understanding of tablet ejection force in pharmaceutical manufacturing appeared to be needed. Since it is routinely measured during tableting machine operations and easily assessed without additional equipment in tablet manufacturing, ejection pressure was selected as a feasible tool for tracking internal failure and mechanical change within BLTs before complete delamination and/or capping in downstream processing.

Thus, this study aimed to evaluate the ejection pressure and the correlation of the findings with the occurrence of internal cracks and delamination within BLTs. Then, a simulation of the compression and ejection processes was provided to confirm the internal crack and delamination mechanisms using the finite element method (FEM). Most studies with respect to tablet failure have been conducted using common single- or bi-pharmaceutical excipients which cannot represent actual pharmaceutical formulations, especially wet granulation methods. Moreover, to the best of our knowledge, the relationship between ejection pressure and internal cracking at the interface of two layers and numerical modeling of tablet failure mechanisms have not been reported for BLTs. Thus, metformin HCl (MF) and evogliptin tartrate (EG) FDC tablets were employed as a model product, currently prescribed for patients with type 2 diabetes mellitus [19,20,21]. In this study, the ejection pressure and difference in diameter were evaluated depending on the main compression pressures (MAIN-Ps). The compaction force required to break MF/EG BLTs between the adjacent MF and EG layers with different MAIN-Ps was also determined. Morphological analysis of the internal cracks at the interface of the BLTs was conducted with different MAIN-Ps using scanning electron microscopy (SEM) observation and non-destructive X-ray microcomputed tomography scans. Then, FEM modeling was performed to simulate the compression and ejection processes of the BLTs to confirm the internal crack and delamination mechanisms with different MAIN-Ps. Furthermore, the modeling results were compared with the experimental results to ensure the consistency of the FEM model.

## 2. Results and Discussion

### 2.1. Effect of MAIN-P on Porosity and Compaction Breaking Force of MF/EG BLTs

In a previous study, the properties of wet granulation were evaluated [22]. To evaluate the effects of the MAIN-P on the ejection pressure, compaction breaking force, and porosity of the tablets, the morphology, particle size, density, and flowability of wet granulation were determined. In order to prove it was suitable for granule compaction, flowability was determined to be “excellent” or “good” by using Hausner’s ratio (HR) and Carr’s index (CI) values of MF- and EG-loaded granules. Subsequently, the free-flowing granules were employed for the production of MF/EG BLTs to assess the ejection pressure and detect internal cracks.

The integrity of tablets depends on their ability to establish inter-particulate bonds while undergoing compression in the die and the ability of these bonds to withstand elastic expansion from the die [23]. The USP reference standards provide guidelines and standards to assess the integrity of pharmaceutical tablets, including porosity, tablet compaction breaking force, friability, and disintegration [24]. Among these factors, porosity and breaking force tests are routinely carried out for in-process quality control and monitoring [25] and have been commonly employed for predicting the performance of tablets, including their disintegration, dissolution, and mechanical integrity [26]. Therefore, to determine whether it is possible to track micro-cracks within BLTs that degrade mechanical integrity, the porosity and the compaction breaking force were evaluated, with MAIN-Ps varying and the pre-compression pressure (PRE-P) fixed at 40 MPa.

**Figure 1:**
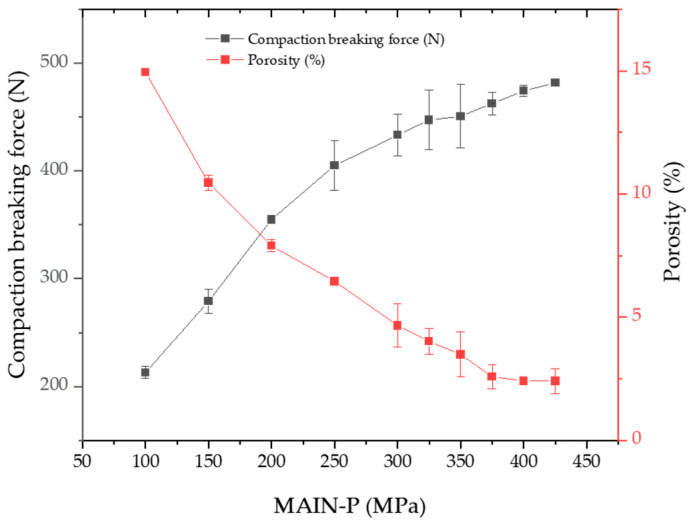
Effect of MAIN-P on the porosity and compaction breaking force of MF/EG BLTs. Porosity and compaction breaking force of BLTs dependent on different MAIN-Ps. Note: PRE-P was fixed at 40 MPa, while MAIN-P was varied from 100 to 425 MPa during compression.

To determine the porosity of the BLTs, a helium Ultrapyc 1220e Automatic Gas Pycnometer (Anton Parr, Graz, Austria) was employed. As depicted in Figure 1, the porosity of the MF/EG BLTs exhibited a linear decrease with increasing MAIN-P. Moreover, the porosity of the BLTs decreased from 14.95 to 2.80% as the MAIN-P increased from 100 to 350 MPa. A decrease in porosity was not observed for BLTs above 375 MPa. As the compression pressure increases, the elastic and plastic deformation of particles becomes dominant [24]. Subsequent increases in compression pressure could lead to particle fragmentation, consequently inducing further densification and a reduction in porosity in the powder. The reduced distance between the particles could result in intermolecular forces, such as Van der Waals forces [27]. Consequently, increasing the MAIN-P enhanced the mechanical integrity of the MF/EG BLTs [28]. 

As the MAIN-P increased from 100 to 200 and 300 MPa, the compaction force needed to induce delamination in the BLT linearly increased to 213, 355, and 433 N, respectively. Then, the compaction breaking force continued to increase from 463 to 481 N without a noticeable change in the linear trend as the MAIN-P increased from 375 to 425 MPa, the compression pressure at which micro-cracks within the tablets were expected to be present. Based on these findings, we concluded that tracking tablet failure related to local micro-stress states is unlikely using traditional testing methods, such as the USP reference standards, including porosity and compaction breaking force tests. Particularly in multi-layered formulations, an emerging technology characterized by efficiency and high demand, USP standard testing methods are insufficient for tracking interface micro-cracks. Thus, novel quality control tools are required to meet current quality expectations and requirements set by regulatory agencies.

### 2.2. Evaluation of Crack Occurrence of MF/EG BLTs Depending on MAIN-P

**Figure 2:**
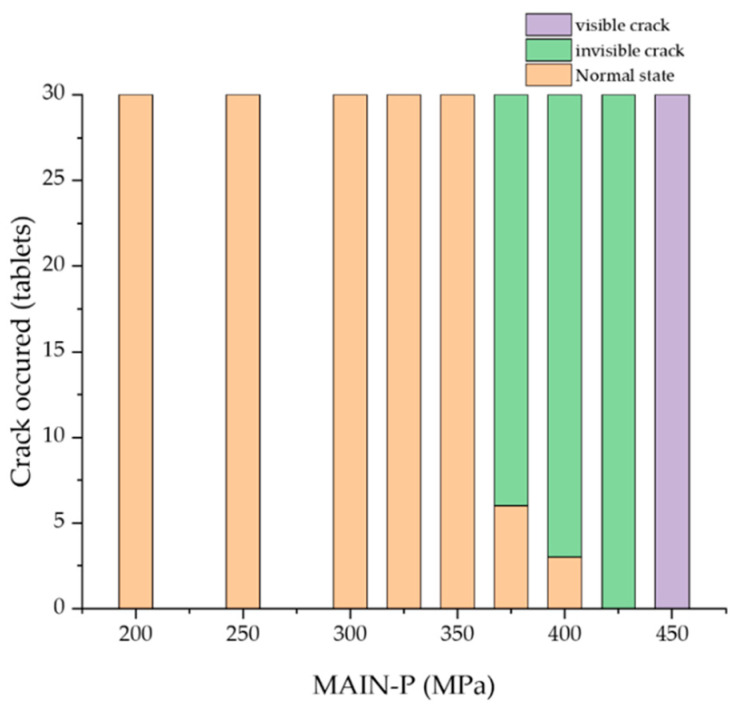
Evaluation of crack occurrence during the compression of BLTs depending on MAIN-P. Notes: PRE-P was fixed at 40 MPa, and the MAIN-P was varied within the range of 200 MPa to 450 MPa during compression. To determine internal cracks, the cross-sectional surface of the BLT was observed by cutting perpendicular to the interface after the main compression. Data are expressed as the number of cracked tablets out of the total compressed tablets (*n* = 30).

BLTs with different MAIN-Ps were macroscopically observed after cross-section procedures to scrutinize invisible cracks in tablets. In this study, two main classes of cracks were discussed to characterize tablet cracks: (i) visible cracks and (ii) micro-cracks, which are invisible from the outside. Herein, the crack occurrence was evaluated during the compression of BLTs with MAIN-Ps ranging from 100 MPa to 450 MPa and with the PRE-P fixed at 40 MPa (Figure 2). The extent of tablet fracture was macroscopically investigated by assessing the presence of cracks in the tablets after the main compression. To identify invisible BLT cracks, the cross-sectional surfaces of the tablets were cut using a razor perpendicular to the interface after complete ejection from the die cavity.

In the experiments, a visible crack separating a tablet into two distinct horizontal layers was observed at 450 MPa of MAIN-P. Interestingly, at 375–425 MPa, an internal crack was observed with a thin layer of the BLT at the end of the ejection. These micro-cracks were located in the center at the interface between the MF and EG layers of the BLT, while the edge remained integrated. These cracks were undetected by external visual examination, which could promote complete delamination. The number of invisible crack occurrences in the BLT increased as the MAIN-P increased from 375 to 425 MPa. However, this crack did not affect the increasing trend in compaction breaking force (Figure 1). This result aligns with a previous report that suggests that micro-cracks might not significantly impact the breaking force of tablets under certain conditions, especially when tablet strength is high [29]. Therefore, we focused on evaluating a tool to track internal crack formation in BLTs at its onset before a critical failure occurs in downstream processing.

### 2.3. SEM Observation of the Internal Structure of MF/EG BLTs

The internal structure of the MF/EG BLTs, depending on the MAIN-P, was further analyzed using SEM observations (MIRA3 LMH, TESCAN, Brno, Czech Republic) to investigate the morphology of internal cracks. The cracks were evaluated upon complete ejection from the die cavity. FE-SEM analysis was performed on a cross-sectional surface of the BLT, cut perpendicular to the interface, after the main compression. 

**Figure 3:**
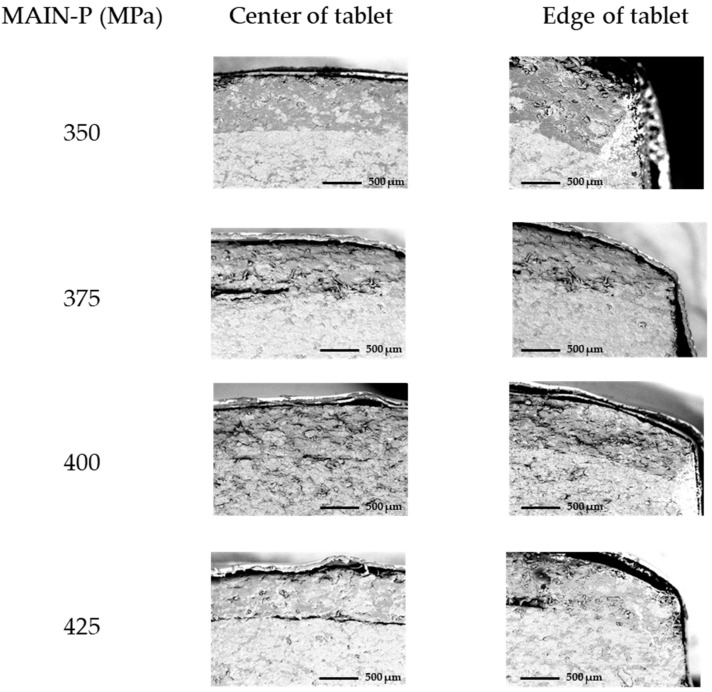
Representative cross-sectional FE-SEM images at the interface of MF/EG BLTs. Analysis of MF/EG BLTs compressed with a 40 MPa PRE-P, with the MAIN-P varying from 350 MPa to 425 MPa during compression. Notes: FE-SEM analysis was performed on a cross-sectional surface at the center (**left**) and edge (**right**) of the BLTs, cut perpendicular to the interface, after the main compression. The samples were coated with platinum and analyzed at 15 kV. Images are ×1000 magnified.

In the main compression at 350 MPa pressure, the cross-sectional interfacial surface was completely integrated mechanically, with no noticeable cracks at either the center or the edge of the BLT (Figure 3). The interface line between each layer was physically hardly distinguished, indicating that each granule might have been deformed and formed inter-particulate bonds during compression. This resulted in the granules interconnecting to form a firm interface. However, when compressed with a higher MAIN-P (375 MPa), a crack was observed at the center of the interface, suggesting that each layer might have been separated due to the high compression pressure. Interestingly, the shape of the interface at the edge of the BLT was preserved with no crack. Similarly, in the BLTs main compressed with 400 MPa and 425 MPa, the center of the interfacial surface showed a crack, while the edge remained integrated. These tablets with internal cracks were undetected by external visual examination, which is consistent with the findings of a previous report that when the part of the tablet near the edge undergoes compression pressure, the cracks initially formed at the center may stop before reaching the tablet edge [30]. These cracks in proximity to the die surface interact with the stress field from the die during ejection, leading to the extension of pre-existing micro-cracks or even delamination [31]. Thus, the possibility of such cracks within a tablet should be considered in the manufacturing process, although they are not immediately detected just after compression.

### 2.4. Non-Destructive X-ray Microcomputed Tomography Analysis of the Internal Structure of MF/EG BLTs

**Figure 4:**
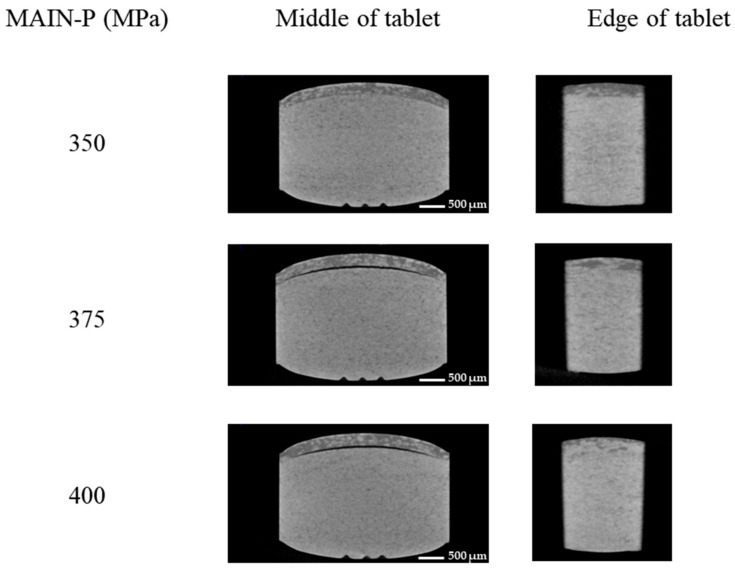
Representative cross-sectional X-ray microcomputed tomography images of MF/EG BLTs. Analysis of MF/EG BLTs compressed with 40 MPa PRE-P and the MAIN-P varying from 350 MPa to 400 MPa during compression. Notes: X-ray microcomputed tomography was performed on cross-sectional surfaces of BLTs after the main compression. The images were taken at the middle (**left**) and end (**right**) cross sections of the specimens. The equipment used was a transmission beam and a tungsten target. The scan parameters were 110 kV, 50 µA, and 5.5 W, respectively, with no filter. The final voxel size was 23 µm.

X-ray microcomputed tomography is a widely employed imaging technique that primarily focuses on identifying and characterizing spatial mass density distributions in compacts; thus, it is suitable for visible and invisible defect tracking [32,33]. To cross-sectionally observe an invisible crack without passive breakage, which may affect the crack extent, the BLT interface was observed using X-ray microcomputed tomography as a non-destructive method after image reconstruction [30]. The X-ray images were taken at the middle and end cross sections of the specimen (Figure 4).

When the PRE-P was compressed to 350 MPa, the shape of the interface between the MG and EG layers was preserved, with no cracks at the middle or the edge. However, at 375 MPa, the BLT appeared to have a crack located approximately in the central plane at the interface of the BLT along the X-direction, whereas no cracks were observed at the edge, consistent with SEM observations. This revealed that central micro-cracks do not propagate to the tablet edge; consequently, the tablet is not completely laminated. The primary finding of this study is that the formation of cracks does not necessarily result in the complete delamination of tablets. Thus, novel quality control tools are required to meet current quality expectations and requirements set by regulatory agencies, and we hypothesized that ejection pressure could be a potential candidate for effectively and efficiently tracking invisible cracks.

### 2.5. Evaluation of the Correlation between Change in Ejection Pressure and Internal Crack Occurrence

A previous study has revealed that high ejection pressure is a risk factor in large batches of tablets, indicating excessive die wall friction, which could result in potential tablet defects [34]. Ejection pressure originates from the constraint of a compressed tablet within a die, exerted by the residual die wall stress along the radial direction. Anuar and Briscoe [17] reported that higher compression stress produces increased initial radial die wall stress in tablets before ejection, consequently causing higher tablet elastic relaxation during ejection. In this process, localized elastic relaxations can lead to fractures occurring in the upper region of the tablet body. Thus, ejection pressure was reported to be a warning indicator that a product is prone to risks such as capping and lamination [18,35]. Given these findings, we hypothesized that invisible internal cracks could be tracked by monitoring ejection pressure. 

**Figure 5:**
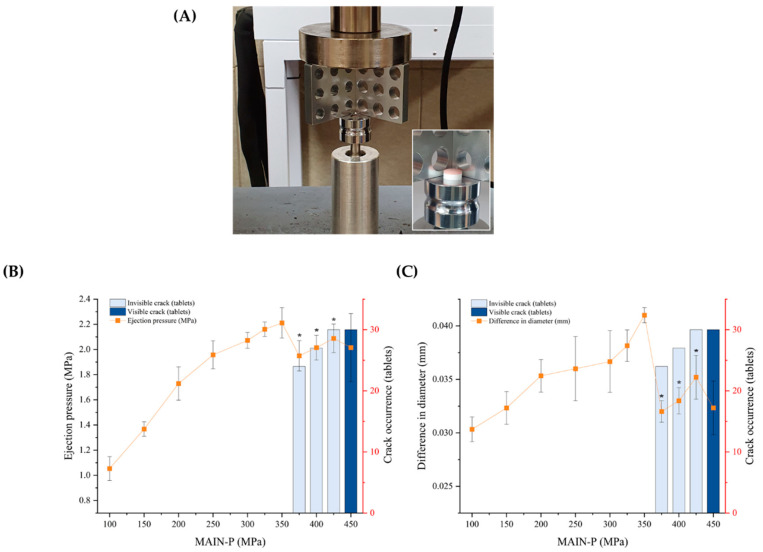
Effect of MAIN-P on the ejection pressure and differences in the diameters of MF/EG BLTs. (**A**) Image of the ejection pressure tester equipped with a commercial Euro standard punch and die. Ejection pressure (**B**) and differences in diameters (**C**) of MF/EG BLTs with different MAIN-Ps. Notes: (**A**) The insert image depicts the BLT after ejection from the die. (**C**) The difference in diameter is expressed as the difference between the initial BLT diameter and the ejected BLT diameter. (**B**,**C**) PRE-P was fixed at 40 MPa, and the MAIN-P was varied from 100 MPa to 450 MPa during compression. Significantly different from ejection pressure (**B**) and difference in diameter (**C**) at 350 MPa (* *p* < 0.05) by the Student’s *t*-test.

The ejection pressure and difference in diameter, depending on the MAIN-P, were evaluated after the tablets were ejected from the die (Figure 5). At first, the ejection pressure was measured with different MAIN-Ps ranging from 100 to 450 MPa, with the PRE-P fixed at 40 MPa. As expected, ejection pressure increased rapidly as the MAIN-P increased from 100 MPa to 350 MPa, and the ejection pressure was determined to be 2.21 MPa at 350 MPa (Figure 5B). Interestingly, despite the MAIN-P increasing from 350 to 375 MPa and an increasing pattern in the compaction breaking force of the BLTs (Figure 1), ejection pressure markedly decreased to 1.95 MPa at 375 MPa, which was statistically different from the ejection pressure at 350 MPa. Similarly, when the BLTs were mainly compressed at 400 MPa and 425 MPa, the ejection pressure was determined to be 2.01 MPa and 2.09 MPa, respectively, remaining in decreased states with values below the ejection pressure at 350 MPa, which is considered the peak point.

The difference in diameter also exhibited an analogous profile (Figure 5C); it increased as the MAIN-P increased to 350 MPa, followed by a steep decrease at 375 MPa. When the MAIN-Ps were 375, 400, and 425 MPa, the difference in diameter was determined to be 0.032, 0.033, and 0.035 mm, respectively. As the compression pressure of the final layer increases, the high stress applied to the tablet produces a higher tablet initial radial die wall stress before ejection, leading to a greater tablet elastic energy during ejection [36]. Accordingly, the BLT, which stored elastic energy in the die, was expanded due to elastic relaxation after being ejected from the die cavity, increasing the diameter of the tablets [17]. However, an internal crack caused by abrupt localized elastic relaxations in the interface of the BLT (the upper part of the body), which is the region of weakest bonding within the BLT, made it challenging to maintain the original shape in the die, resulting in a decrease in the diameter as the tablets fractured [36]. Consequently, the tablet ejection pressure also decreased as the frictional resistance on the die wall weakened [17]. Another significant decrease in ejection pressure and difference in diameter was observed due to complete delamination at a pressure of 450 MPa. Therefore, the precursor symptoms of quality degradation due to invisible cracks are the same as the changes in the initial decreasing pattern of ejection pressure observed below the complete delamination at 450 MPa. Thus, the MAIN-P should be tightly controlled below the initial peak value of ejection pressure at 350 MPa during the compression setting stage to prevent cracks. These findings suggested that internal structural changes and cracks in BLTs could be tracked based on observing abrupt changes in the ejection pressure in the process by measuring the ejection pressure pattern before the manufacturing process.

In tablets, cracks in the final product could go undetected if controlled only with traditional testing methods, such as the USP reference standards, which include friability, fracturing, or breaking force testing [8]. As an alternative method, X-ray microcomputed tomography can be employed as a non-destructive and material-sparing approach with advantages for analytical studies and defect characterization applications, while most of the pharmacopeial techniques are semi-destructive or invasive to the samples. However, these alternative methods are time-consuming and do not permit a quantitative evaluation of the factors influencing failure. Thus, in this study, our findings suggest that ejection pressure can be a tracking indicator to prevent internal crack formation at the interface in the BLT manufacturing process. Moreover, interpreting the internal crack and delamination mechanism of BLTs only from the experimental data is challenging. Hence, to aid consolidating and interpreting these results, FEM simulation was employed.

### 2.6. FEM Analysis of Internal Cracks during the Compression Process of MF/EG BLTs

In the pharmaceutical industry, the FEM has been introduced to better understand tablet compaction behavior. Wu et al. determined, using the FEM, that micro-cracks tend to form during the unloading process in monolithic tablets [27]. Wu et al. also noted that the intensive shear bands that develop during unloading are one of the factors responsible for the occurrence of capping. Mazel et al. used simulations with the FEM to suggest a delamination mechanism for monolithic tablets, indicating that tablet fracture is promoted by tensile stresses localized at the center of the tablet [30]. These stresses, induced by residual die wall pressure and the tablet’s shape, can exacerbate fracture formation with a small band thickness and high compression pressure. Based on these findings, the crack mechanism of the BLT during the compaction process was predicted to be associated with intensive shear bands under high pressures and was considered similar to that of the monolithic tablet.

**Figure 6:**
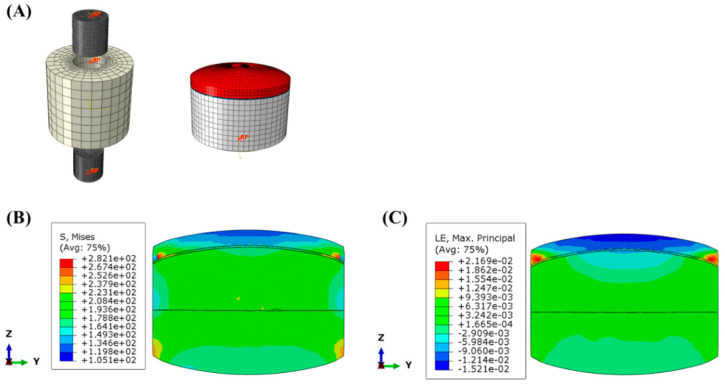
The modeling of die, punch, and distribution of the (**A**) Von Mises stress and (**B**) strain of MF/EG BLTs at the end of the compression. Notes: (**A**) The MF layers were colored white, and the EG layers were colored red. (**B**,**C**) A pressure of 300 MPa was applied as MAIN-P to the BLT in the compression simulation.

The Von Mises stress and strain distribution were analyzed within the tablet at the end of the compression with 300 MPa of MAIN-P to identify the internal stress responsible for the tablet failure using a linear elastic model and axisymmetric simulation conditions. The case in this section corresponds to an internal crack formation during compression in a plane perpendicular to the direction of compression, occurring approximately at the center of the tablet band. Prior knowledge indicates that air is frequently trapped within the formulation granules and particles during compression [37,38,39,40]. Suppose air within the pores fails to escape and becomes compressed within the BLT, its re-expansion during decompression/relaxation could give rise to internal cracks, potentially leading to delamination. In this study, numerical analysis mainly focused on the stress distribution during the compression to understand the reasons that the edges of the BLT are not affected by air entrapment, which contributes to the cracking, as shown in Figure 4. Thus, the first step was to investigate the distributions of Von Mises stress and strain at the end of the compression in the FEM simulation. As shown in Figure 6B, low-stress areas at the center of the BLT interface indicate minimum values (indicated in green), suggesting a loose bond in the MF/EG layer. The entrapment of air within the pores at the BLT interface during the compression phase can expand, thus exacerbating the cracking at the interfaces between layers where the bonds are weak [41,42]. Additionally, the tensile stress along the Z-axis, which pulls the EG layer by vacuum in the die for a short period due to the rapid compression speed of the upper punch during the decompression process, also contributes to exacerbated crack formation. Conversely, in the simulation, high stresses (indicated in red) are concentrated at the BLT interface’s edge, where it contacts the die. As shown in the results, during main compression, the stress increased owing to the die wall pressure from the edge of the tablet in contact with the die. Thus, this stress decreased intra-granular pores and increased plastic deformation at the edge of the BLT [43], making it difficult for air to become entrapped, thereby promoting a tight bond in the MF/EG layer. Similar to the stress distribution, the strain distribution results shown in Figure 6C reveal severe strain around the edge of the BLT. Accordingly, the stress analysis indicates that the internal crack formation considered in this study could be attributed to the concentrated stress at the edge of the interface of the BLT by main compression, leading to firm bonding despite tensile stresses from air entrapment and a vacuum in the die. As a result, crack formation occurred solely at the tablet center, which coincides with the observations using SEM and X-ray microcomputed tomography shown in Figure 3 and Figure 4.

### 2.7. FEM Analysis of Delamination during the Ejection Process of MF/EG BLTs

The numerical results in Section 2.6 identified a mechanism that could lead to internal cracks in the BLT during the compression process. The presence of cracks within the BLT contributes to crack expansion during the ejection of the BLT from the die due to excessive friction between the edge of the BLT and the die surface, finally leading to the delamination of the tablet when ejected [31]. In this section, shear stress distribution during the ejection process was focused on understanding its contribution to delamination in BLTs, followed by a comparison with experimental results. 

**Figure 7:**
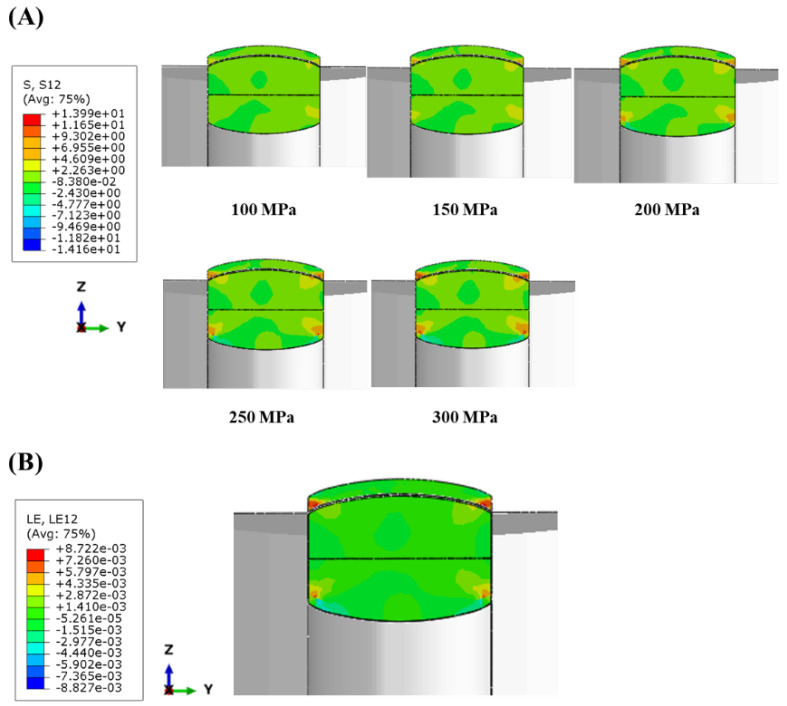
The distribution of shear stress (**A**) and strain (**B**) of MF/EG BLTs in the ejection process. Notes: (**A**) Shear stress distribution of BLTs with different MAIN-Ps from 100 to 300 MPa at the end of the ejection process. (**B**) A pressure of 300 MPa was applied as the MAIN-P to the BLT.

Figure 7A shows the distribution of shear stress within the tablet during the ejection process, aiming to identify the residual stress as the BLT is partially ejected beyond a specified distance from the die. To test the influence of the MAIN-P, five symmetrical pressures were progressively applied (100, 150, 200, 250, and 300 MPa). In the BLT compressed at 200 MPa, the shear stress distribution at the end of ejection reveals maximum values at the top tablet edge, particularly at the MF and EG layer interfaces. As the ejection progresses, the volume of the EG layer ejecting from the die expands due to elastic recovery, whereas the internal MF layer remains compressed within the die [44]. This difference in volume causes severe deformation (Figure 7B) and increased shear stress. The distribution of shear stress observed in MF/EG BLTs is consistent with previous reports on monolithic tablets [45]. The creation of a narrow band with localized and intensive shear stresses, contributing to the upper edge of the monolithic tablet, is associated with promoting tablet failure tendencies. Furthermore, the maximum shear stress exhibited a lower value at 100 MPa (Figure 7A, upper left panel). It increased at the BLT interface as the MAIN-P increased (Figure 7A, lower right panel). These results suggest that initial internal cracks might remain unchanged at low MAIN-Ps without propagating to the edge area. However, suppose the shear stress at the interface edge exceeds the bonding strength of the two layers within the BLT, which are the regions of weakest bonding at high MAIN-Ps. In that case, complete delamination is expected to occur. In conclusion, the complete delamination at the interface of the BLT can be attributed to the gradual ejection process that increases radial pressure and the concentration of shear stress at the interface between the MF and EG layers. 

### 2.8. Quantitative Comparative Study of Ejection Pressure between Experimental and FEM Analyses

**Figure 8:**
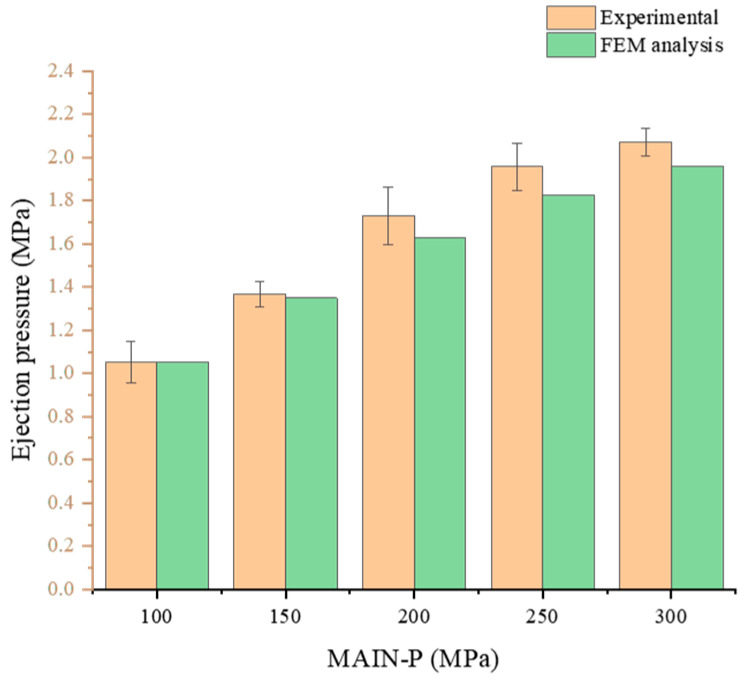
The quantitative comparison of ejection pressure between experimental and predicted results using FEM analysis. Note: Experimental results for ejection pressure were obtained from test results in Figure 5B.

Figure 8 shows a comparison of the ejection pressure results with different MAIN-Ps between the modeling and experimental data, as obtained from Figure 5B, to validate the FEM simulation. The simulation predicted results show that the ejection pressure of the tablets increased linearly as MAIN-P increased and that the values are practically identical to the experimental results, with a difference of less than 10%. These results are consistent with previous reports that a higher compression pressure results in a higher initial radial die wall stress in the tablet before ejection [17]. Consequently, this stress increases tablet elastic relaxation during ejection, impeding movement inside the die and resulting in higher ejection pressure. Based on these comparative analyses, in analytical studies, the ejection pressure can be determined using a numerical model without relying on experimental results, even with changes in factors that affect the ejection pressure. Based on this, a numerical method was suggested to identify fracture patterns in BLTs as an alternative to empirical approaches for practical applications.

## 3. Materials and Methods

### 3.1. Materials

MF (purity over 99 *w*/*w*%, median diameter of 38.33 μm) and EG drug powders (purity over 99 *w*/*w*%, median diameter of 44.74 μm) were obtained from Granules India Limited (Madhapur, Hyderabad, India) and Dong-A ST (Seoul, Republic of Korea), respectively. Polyvinylpyrrolidone (PVP K30) was purchased from BASF (Ludwigshafen Land, Rheinland, Pfalz, Germany). High viscosity grades of HPMC2208 and methacrylic acid copolymers (Eudragit S100) were obtained from Dow Chemical (Montgomeryville, PA, USA) and Evonik (Essen, NRW, Germany), respectively. Carbomer 934P (Carbopol^®^ 934P-NF) and mannitol (Pearlitol 100 SD) were obtained from BF-Goodrich (Cleveland, OH, USA) and Roquette (Lestrem, Pas de Calais, France), respectively. Low viscosity grades of low-substituted hydroxypropyl cellulose (L-HPC) and medium viscosity grades of hydroxypropyl cellulose (Klucel) were obtained from Shin-Etsu Chemical (Otemachi, Chiyoda-ku, Japan) and Ashland (Wilmington, DE, USA), respectively. Pregelatinized starch (Starch 1500) and croscarmellose sodium (Ac-Di-Sol) were supplied by Colorcon (Harleysville, PA, USA) and FMC Corp. (Philadelphia, PA, USA), respectively. Colloidal silicon dioxide (Aeroperl 300) was purchased from Evonik (Essen, Germany). Magnesium stearate and iron oxide (Fe_2_O_3_) were acquired from FACI Asia Pacific (Merlimau Pl., Jurong Island, Singapore) and Univar (Billerica, Essex, UK).

### 3.2. Preparation of MF and EG Granules Using the Wet Granulation Method

Both MF- and EG-loaded granules were prepared using wet granulation for a batch of 300,000 tablets, as previously described [46]. The exact composition of the mixtures subjected to granulation processes is described in a previous study [22]. MF-loaded granules were prepared by dissolving binders in an ethanol–water solution, spraying them onto the drug powder, and mixing them with excipients, followed by drying and sieving. EG-loaded granules were similarly processed, with specific mixing and drying parameters, before adding disintegrants and lubricants for final preparation.

### 3.3. Preparation of MF/EG-Loaded BLTs

**Figure 9:**
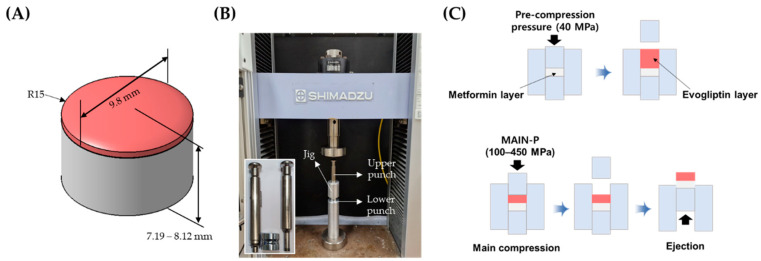
Schematic illustration of MF/EG BLT and fabrication process. (**A**) MF/EG BLT. (**B**) Universal testing machine for compaction of granules. (**C**) Fabrication processes of MF/EG BLT. Note: (**B**) The insert image depicts the punch and die in the jig.

As previously described, MF/EG-loaded BLTs were fabricated by sequential compression of EG- and MF-loaded granules [22] (Figure 9). For the preparation of the BLTs, the MF-loaded granules were manually filled into a die. The granules were pre-compressed using a universal testing machine (JP/AG-50kNX, Shimadzu, Kyoto, Japan) with a newly designed jig that held the punch and die at 40 MPa of PRE-P to prepare the first tablet layer. The testing machine was equipped with a commercial Euro standard D441 tool punch and a round-shaped, convex-type die with a diameter of 9.8 mm and a thickness ranging from 7.19 to 8.12 mm.

Subsequently, the EG-loaded granules were added to the first layer (the MF layer) and compressed at a MAIN-P ranging from 100 to 450 MPa. The MF layers were colored white, and the EG layers were colored red. Subsequently, the tablets were ejected from the die by pushing the first layer upwards with a punch. The compression and ejection speeds were set to 200 and 200 mm/min, respectively. The definitive diameter of the BLT was measured with a digital micrometer (Mitutoyo 395-251, Mitutoyo, Tokyo, Japan) immediately after ejection. The difference in diameter was calculated as the difference between the initial BLT’s diameter and the ejected BLT’s diameter.

### 3.4. Porosity of MF/EG-Loaded BLTs Dependent on Compression Pressure

To determine the porosity (ε) of BLTs compressed with varying PRE-Ps and MAIN-Ps, true density was determined by measuring the true volume of the sample through a helium Ultrapyc 1220e Automatic Gas Pycnometer(Anton Parr, Graz, Austria), as previously described [22]. The porosity (*ε*) was then calculated using the following Equation:

(1) $$\varepsilon =(1-\frac{m}{\rho_{t}v})\times 100$$

where *ρ_t_* is the true density, *m* is the weight of the tablet, and *v* is the volume of the tablet. The apparent volume of the tablet was calculated utilizing a 3D modeling program (CATIA V5R21, Dassault Systemes, Vélizy-Villacoublay, France). 

### 3.5. Compaction Force Required to Break MF/EG-Loaded BLTs

The compaction force needed to crush or delaminate MF/EG-loaded BLTs was assessed using a universal testing machine (JP/AG-50kNX; Shimadzu, Kyoto, Japan), as previously described [22]. The prepared BLTs were positioned between two plates and compressed onto the tablet at a 1 mm/min speed, resulting in fracture or delamination of the BLTs. The peak force obtained from the force–displacement plots was determined as the compaction force.

### 3.6. Ejection Pressure of MF/EG-Loaded BLTs

The tablet ejection pressure was determined using a universal testing machine (JP/AG-50kNX, Shimadzu, Kyoto, Japan). A newly designed apparatus was used to measure the ejection pressure of the BLTs (Figure 5A). The tablets were ejected from the die by pushing the first layer upward with the punch. The ejection speed was set to 200 mm/min. The force and distance were measured and recorded continuously by the sensor until the tablet completely exited the die, and the peak force obtained from the force–displacement plots was determined as the ejection pressure. The BLT, ejected from the die after applying the ejection pressure, is shown in Figure 5A (small box to the right). The tablet ejection pressure (*P*) was calculated as the maximum axial ejection force (*F*) divided by the cross-sectional area (*A*) of the tablet using Equation (2).

(2) $$P=\frac{F}{A}$$

### 3.7. SEM Observation Analysis of the Interface of BLTs

The internal cross section of the BLT was examined using a scanning electron microscope (MIRA3 LMH, TESCAN, Czech Republic). The BLT was cut in a direction perpendicular to the diameter from the first layer to the second layer using a razor. Subsequently, a half tablet was mounted to a copper stub with the surface facing upward, utilizing double-sided carbon tape (Sungho sigma, Suwon, Republic of Korea). The sample was then coated with a thin layer of Pt. Microphotographs of the coated samples were obtained using scanning electron microscopy at an acceleration voltage of 15 kV.

### 3.8. X-ray Microcomputed Tomography

The specimens were scanned using X-ray microcomputed tomography (FF35CT, YXLON, Hamburg, Germany). The facility used a transmission beam and a tungsten target. The scan parameters as the conditions at the source for generating X-rays were 110 kV, 50 µA, and 5.5 W, with no filter. The final voxel size was 23 µm. A total of 3600 projections for a 360° rotation were recorded with a binning factor of 1 and an exposure time of 151 ms, resulting in a total scanning time of 9 h. Tomographic reconstructions were performed using VGstudio MAX software (Volume Graphics https://www.volumegraphics.com/en/products/vgsm.html#, Heidelberg, Germany) with default parameters.

### 3.9. FEM Simulation

The modeling was performed using CATIA software (CATIA V5R21, Dassault Systemes, Vélizy-Villacoublay, France). For the FEM simulations to analyze tablet behavior during the tablet compaction process, Abaqus^®^ software (Abaqus/CAE 2022, Dassault Systèmes, Vélizy-Villacoublay, France) was employed, which is commonly used for numerical modeling. In simulation, a biconvex BLT model was adopted with a high-density mesh, which is much more complicated than that of a flat tablet [47]. A schematic representation of the obtained rigid punch, die, and then the BLT, assuming isotropic material with linear elastic behavior, is shown in Figure 6A. The Young’s modulus and Poisson’s ratio for the BLT were determined from the BLT strength test results (Figure 1). A rigid lower punch was fixed, and uniform compressing pressures of 100, 150, 200, 250, and 300 MPa were applied to the tablet in a diametrically perpendicular direction to obtain comparable results to those performed experimentally. A static friction coefficient of 0.20 was determined for the contact between the BLT and the metallic surfaces in a previous publication [45]. Subsequently, the lower punch was moved upwards by applying a displacement boundary condition to eject the tablets. During ejection from the die, the shear stress and strain distribution of the BLT were analyzed with the FEM model.

### 3.10. Statistical Analysis

Each experiment was performed at least three times, and the data are presented as the means ± standard deviations (SDs). Statistical significance was analyzed using the Student’s *t*-test, and *p* < 0.05 was considered significant using Minitab software (version 21.1, State College, PA, USA).

## 4. Conclusions

The study comprehensively evaluated the relationship between ejection pressure and MAIN-P, aiming to identify its relationship with the internal crack formation of MF and EG BLTs. Subsequently, FEM analysis provided a comprehensive understanding of the tablet failure mechanism in MF/EG BLTs. An abrupt decrease in ejection pressure was observed with the development of internal cracks at higher pressures (375–425 MPa), despite the absence of a noticeable pattern in compaction breaking force. The FEM simulation successfully confirmed the mechanism of tablet failure, including invisible cracks and delamination. These findings indicate that, in the manufacture of BLTs, ejection pressure can be employed as a non-destructive and easy-to-monitor method for preventing internal cracks. This is based on observing abrupt changes in the ejection pressure pattern before the manufacturing process. Moreover, the simulations provide plausible mechanisms of tablet failure in BLTs, aiding in identifying potential structural weaknesses or failure points in tablets.

## Data Availability

Data is contained within the article.
